# A rare pulmonary epithelioid angiosarcoma with ALK rearrangement: a case report and literature review

**DOI:** 10.3389/fonc.2026.1780353

**Published:** 2026-02-26

**Authors:** Jiuyu Gong, Li Zheng, Fangfang Tian, Peixin Xiao, Dong Yu, Lin Jiang, Pengtao Bao

**Affiliations:** 1 Hubei Province Corps Hospital of Chinese People's Armed Police Force, Wuhan, China; 2 The Eighth Medical Center of Chinese People's Liberation Army General Hospital, College of Pulmonary and Critical Care Medicine, Chinese PLA General Hospital, Beijing, China

**Keywords:** ALK, epithelioid angiosarcoma, NGS - next generation sequencing, pulmonary disease, treatment strategy

## Abstract

Epithelioid angiosarcoma is a rare type of malignant tumor that progresses rapidly and currently lacks standard and effective treatment methods. We present herein the first reported case of rare pulmonary epithelioid angiosarcoma harboring an *EML4-ALK* fusion, in which targeted therapy demonstrated efficacy. This advanced, unresectable epithelioid angiosarcoma continued to progress despite prior treatments, including chemotherapy, anti-angiogenic therapy, immunotherapy, and radioactive particle implantation. Given the absence of standardized treatment protocols for this malignancy, we performed next-generation sequencing (NGS) to identify potential therapeutic targets, which revealed an *ALK* fusion. Subsequent *ALK*-targeted therapy proved effective, providing novel therapeutic insights for patients with advanced, unresectable disease. Further studies are warranted to elucidate the mutational landscape of pulmonary epithelioid angiosarcoma and its implications for disease pathogenesis, progression, treatment response, and prognosis.

## Introduction

Epithelioid angiosarcoma (EA) is a rare subtype of angiosarcoma, characterized as a highly aggressive vascular mesenchymal malignancy that predominantly affects the skin and deep soft tissues. The clinical manifestations of EA are often nonspecific, and its imaging features also lack sufficient diagnostic specificity, often leading to misdiagnosis as metastatic carcinoma, tuberculosis, or other diseases (1). Histologically, EA is distinguished by atypical epithelioid endothelial cells, and immunohistochemical analysis confirms the expression of vascular endothelial markers such as CD31 and CD34. To date, no standardized treatment guideline has been established for EA. For localized lesions, surgical resection remains the primary therapeutic option. However, for the cases which surgery is not feasible or insufficient, alternative treatments such as chemotherapy, anti-angiogenic agents, and immunotherapy have been investigated, though their overall efficacy remains limited (2). Although a few patients underwent gene sequencing and mutations in targeted genes were identified, few patients benefited from targeted therapy. Herein we report an EA patient with *ALK* rearrangement who responded to targeted therapy, indicating that next-generation sequencing (NGS) technology applied in rare carcinoma has significant clinical implications for guiding treatment decisions.

## Case presentation

**Figure 1:**
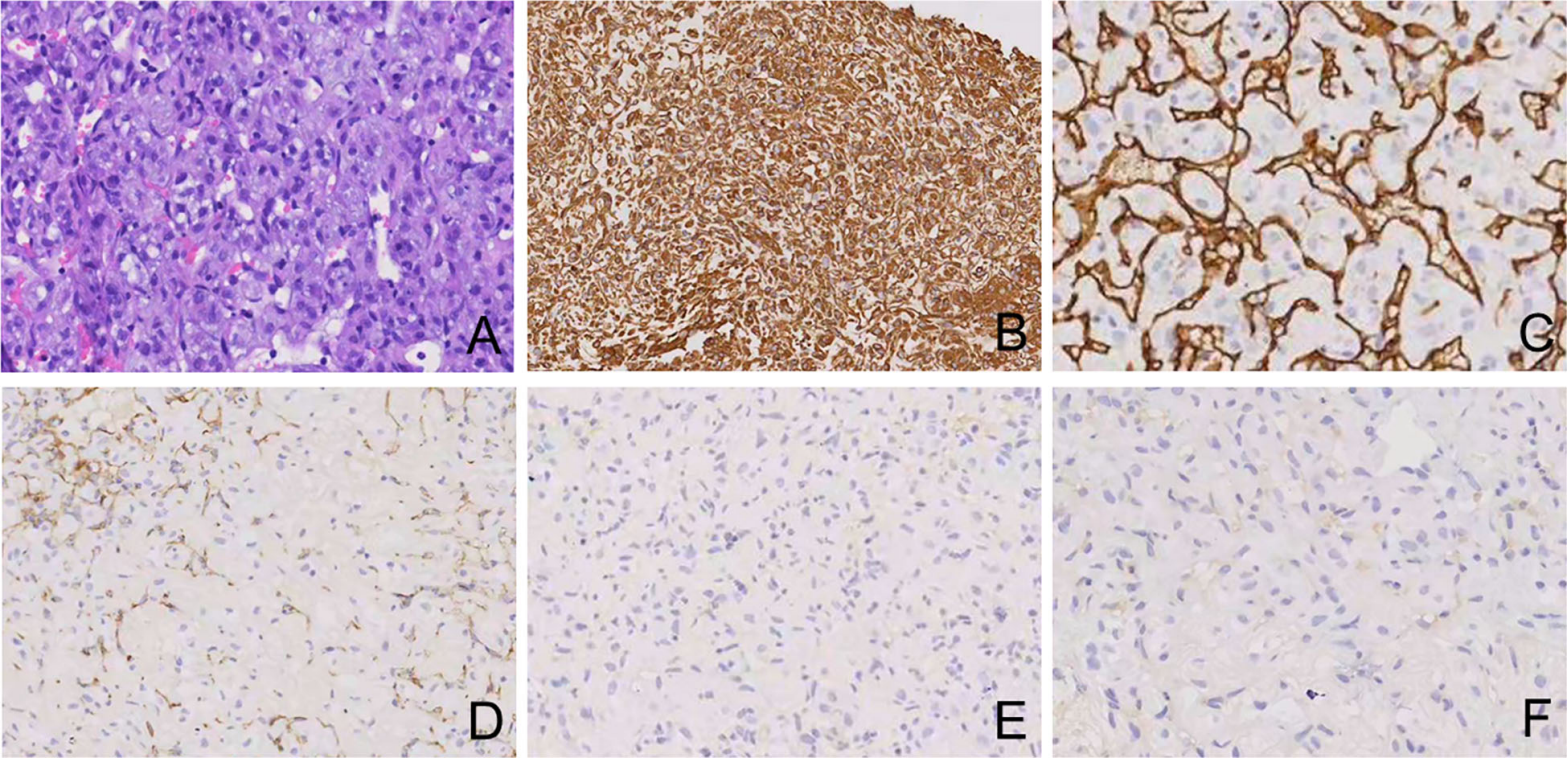
Histologic features **(A-F)** of PEA with HE stains and immunohistochemistry. **(A)** HE, 200×; **(B)** Vimentin, 100×; **(C)** CD34, 200×; **(D)** CD31, 100×; **(E)** Desmin, 100×; **(F)** Napsin A, 200×.

A 65-year-old man presented to hospital on June 6, 2024, with chest pain. The patient had a history of type 2 diabetes mellitus, carotid artery plaque, mild smoking, and an allergy to penicillin drugs. His sister had passed away due to breast cancer. The chest computer tomography (CT) revealed minimal bilateral pulmonary inflammation and multiple subpleural nodules in the left lung; no further therapeutic interventions were undertaken at that time. On June 20, 2024, a follow-up chest CT demonstrated multiple nodules and masses in the left lung, which had increased in size compared to before. Additionally, a small amount of pleural effusion was noted. On June 26, a CT-guided percutaneous lung biopsy was performed. Immunohistochemical analysis (**Figure 1**) revealed the following results: CD31 (+), CD34 (+), Cytokeratin (+), VIMENTIN (+), EMA (partially positive), F8R (+), Fli-1 (+), SMA (+), Ki-67 (>75% positivity), S-100 (focal +), Desmin (-), Napsin A (-), P40 (-), P63 (-), TTF-1 (-). Hematoxylin and eosin (HE) staining showed tumor cells arranged in cord-like and trabecular patterns. The tumor cells exhibited an epithelioid morphology, with abundant cytoplasm, oval nuclei, prominent nucleoli, and frequent mitotic figures. Rich blood sinuses were observed within the stroma. Based on the immunohistochemical profile and histological findings, the diagnosis was consistent with epithelioid angiosarcoma.

To further assess the condition, the patient underwent a positron emission tomography (PET) scan, which revealed multiple masses and nodules beneath the pleura with increased fluorodeoxyglucose(FDG)uptake, indicative of a potential malignant tumor. Additionally, nodules were observed in the intercostal muscle space between the 7th and 8th ribs on the left side, along with metastatic lesions in the left femur. Due to the presence of multiple metastases, the patient was deemed unsuitable for surgical resection. Based on previous case reports, we performed chemoembolization of the local lesion using a regimen of 60 mg cisplatin and 80 mg epirubicin delivered via the intercostal artery, while concurrently administering oral anlotinib. Anlotinib is a multi-target tyrosine kinase inhibitor (TKI) independently developed in China, which has been approved for the treatment of five cancer types, including non-small cell lung cancer and soft tissue sarcoma. It primarily targets vascular endothelial growth factor receptors 1/2/3 (VEGFR1/2/3), platelet-derived growth factor receptors α/β (PDGFRα/β), fibroblast growth factor receptors 1–4 (FGFR1–4), and c-Kit, exerting broad antiangiogenic effects and direct antitumor activity (3, 4). However, acquired resistance to anlotinib represents a significant clinical challenge. To overcome resistance and enhance therapeutic efficacy, anlotinib is frequently combined with other treatment. Notably, the combination of anlotinib with radiotherapy and immunotherapy has demonstrated synergistic antitumor immune effects in non-small cell lung cancer (NSCLC) (5). Additionally, in the first-line treatment of soft tissue sarcoma (STS), anlotinib combined with epirubicin followed by maintenance therapy has shown promising clinical outcomes (3).

On July 22, a follow-up chest CT scan revealed that the masses and nodules had increased in size compared to previous imaging, suggesting treatment ineffectiveness and disease progression. Consequently, radionuclide interstitial particle implantation therapy was administered on July 23, while anlotinib and incadronate disodium treatments were continued. On September 10, a subsequent chest CT review indicated that some lesions demonstrated changes consistent with the effects of particle implantation, whereas other lesions continued to progress. On September 11, the patient underwent combination therapy comprising 300 mg albumin-bound paclitaxel, 600 mg bevacizumab, and 200 mg tislelizumab for anti-angiogenic and immunotherapeutic purposes. Additionally, incadronate disodium was re-administered to address bone metastasis management. Owing to adequate prophylactic interventions—specifically antiemetic prophylaxis, gastric protection, and nutritional supplementation—the patient exhibited merely mild gastrointestinal intolerance (GradeI) during chemotherapy, without evidence of severe or unexpected adverse events.

**Figure 2:**
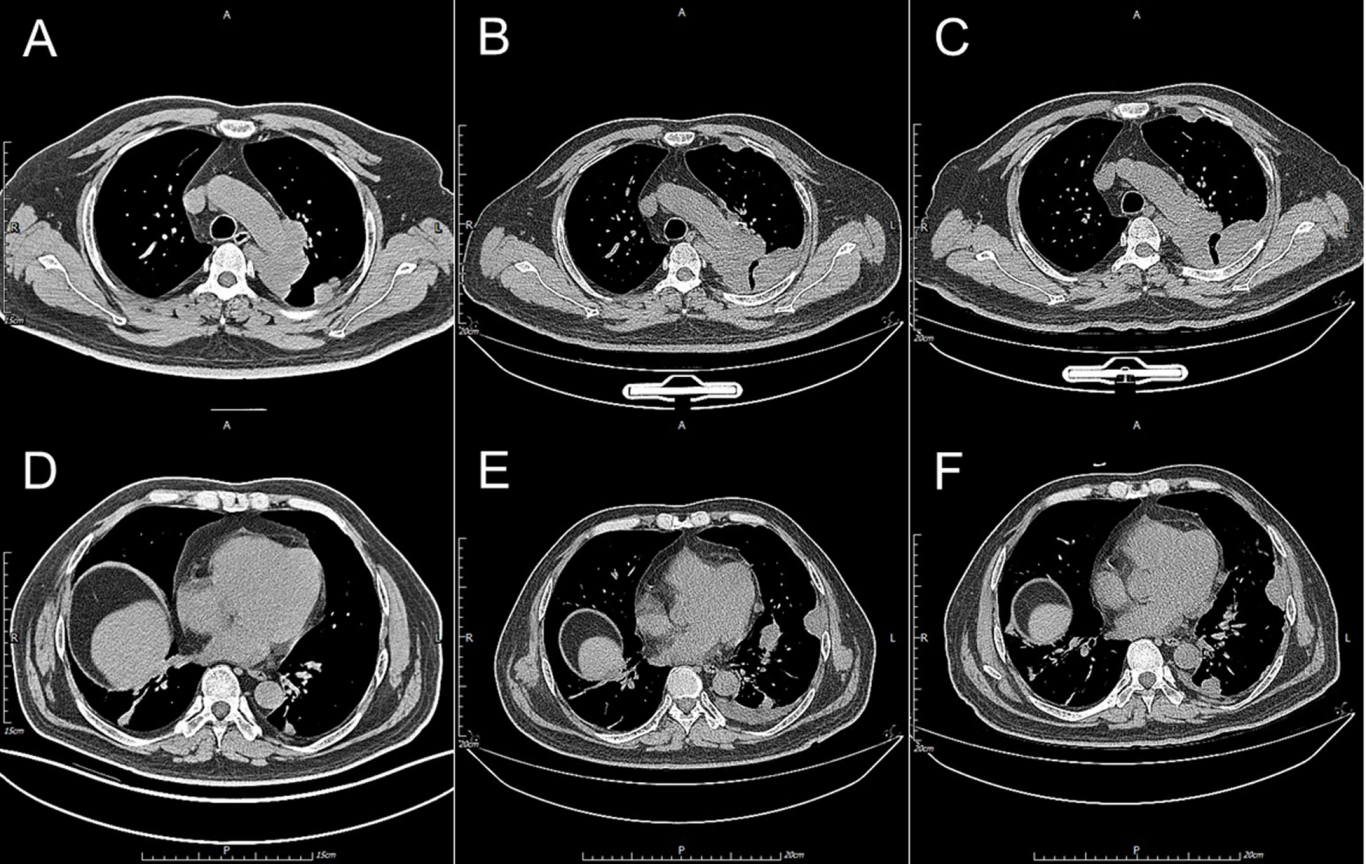
**(A)** CT scan of the patient with PEA throughout his treatment is presented. Images **(A, D)** acquired on June 22nd, 2024, demonstrate a mass adjacent to the right pulmonary hilum with evidence of pleural invasion. Despite undergoing a series of treatments, the mass exhibited rapid growth over a two-month period [images **(B, E)**]. Notably, following the initiation of ALK-targeted therapy for 2 months, the tumor demonstrated disease stabilization and partial regression [images **(C, F)**].

**Figure 3:**
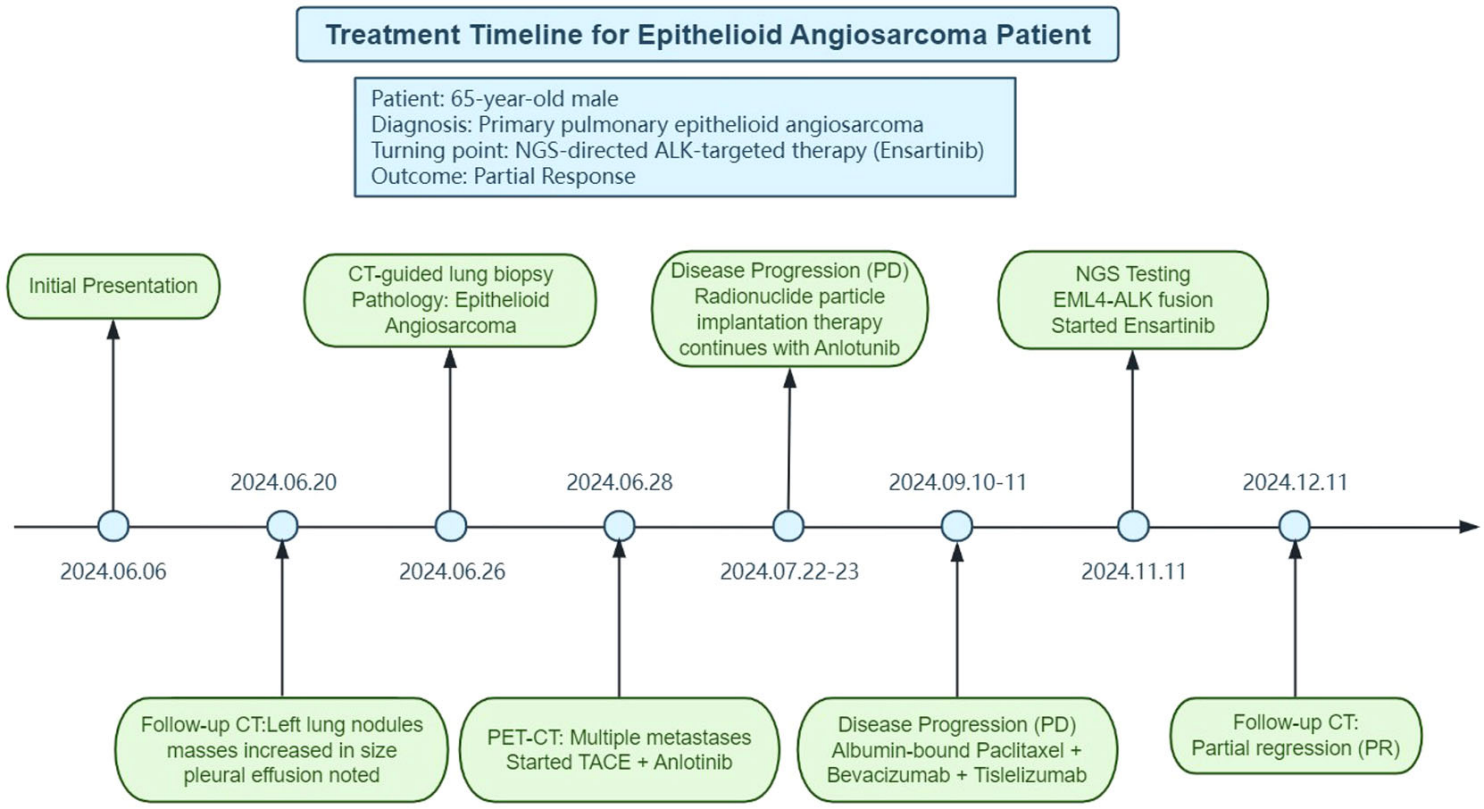
Treatment timeline of the pulmonary epithelioid angiosarcoma patient.

On November 11, due to the ongoing progression of the disease, next-generation sequencing (NGS) using the Geneplus OncoScreen Plus (Tissue) panel was performed on a paraffin-embedded tumor tissue to comprehensively detect four types of genetic alterations among 1066 tumor-related genes. This panel employs target capture and ultra-deep sequencing on a high-throughput platform, encompassing: (i) entire exonic regions of 407 genes; (ii) intronic regions, promoters, and fusion breakpoints of 49 genes; and (iii) coding regions of 656 genes. For plasma cell-free DNA (cfDNA) analysis, the proprietary ER-Seq technology developed by Geneplus Medical Laboratory was additionally applied. The panel enables comprehensive detection of four major variant classes, including single nucleotide variants (SNVs), small insertions and deletions (indels), copy number alterations (CNAs), and gene fusions. Six genetic alterations were detected, and two of which were potentially actionable for targeted therapies: a mutation in *NF2* (p.Q212) and an *EML4-ALK* fusion (V1 variant) (**Table 1**). Given the presence of *ALK* fusion, along with a low tumor mutational burden (TMB-L) and microsatellite stability (MSS), it was determined that the patient would likely derive minimal benefit from immunotherapy. Considering the established efficacy of *ALK*-targeted therapy in cancers harboring *ALK* mutations, the *ALK* inhibitor ensartinib was selected for treatment. A follow-up chest CT (**Figure 2**) scan conducted one month later demonstrated partial regression of lesions, confirming the effectiveness of the targeted therapy. All the therapeutic timeline was summarized and presented in **Figure 3**.

**Table 1:** Targets associated with tumor gene testing and their corresponding targeted therapeutic agents.

Gene	Variation type	Functional region	Mutaion frequency	Mutation level	Sensitive drugs
*NF2*	Gene fusion (V1)	EX13:EX20	51.7%	Type II	Everolimus (Grade D*, sensitive), Sirolimus (Grade D*, sensitive), Temsirolimus (Grade D*, sensitive)
*EML4-ALK*	p.Q212	EX7	31.4%	Type II	Alectinib (Grade C*, sensitive), Brigatinib (Grade C*, sensitive), Ceritinib (Grade C*, sensitive), Crizotinib (Grade C*, sensitive), Ensartinib (Grade C*, sensitive), Envonalkib (Grade C*, sensitive), Iruplinalkib (Grade C*, sensitive), Lorlatinib (Grade C*, sensitive)

*The evidence levels for the association between genetic variations and drug sensitivity are classified into four tiers according to the cancer variant interpretation guidelines published by AMP/ASCO/CAP (PMID: 27993330). Grade A: Genetic variants have received FDA approval for use in specific cancer types or are included in established professional clinical guidelines. Grade B: Evidence is supported by findings from large-scale clinical studies and has been widely accepted by clinical experts. Grade C: Genetic variants have been approved by the FDA or other recognized professional organizations for use in different cancer types; or are utilized as inclusion criteria in clinical trials; or are supported by multiple small-scale investigations. Grade D: Evidence is derived from preclinical research or limited case observations.

## Discussion

Pulmonary epithelioid angiosarcoma is a rare and highly aggressive malignant tumor, yet no standardized treatment protocol has been established to date. Based on prior case reports, surgical resection has historically been regarded as the primary treatment option for patients with locally confined lesions. However, due to the highly invasive nature of this tumor, most patients are diagnosed with metastatic disease and are thus not candidates for surgery. Currently, chemotherapy, targeted therapy, and immunotherapy remain under investigation, with only limited efficacy reported in some cases (**Table 2**).

**Table 2:** A case review of epithelioid hemangioendothelioma of the lung.

Case	Gender	Age	Symptoms	Pathology	Targets	Treatment	Prognosis
1 (17)	Female	58	cute onset of chest pain, progressive dyspnea, and worsening dysphagia	EMA(+), CD31, ERG(+), TLE1(+), CD9(+), hcaldesmon(+)	Missense *NRAS* mutations and a splice donor site in the *TP53* gene	Palliative radiotherapy	Mortality
2 (2)	Female	35		Multiple vascular neoplasms evolved into epithelioid angiosarcoma nine years later	*EWSR1::NFATC2* gene fusion and *PIK3CA* gene mutation	Recurrence occurred following the surgical resection of bone lesions	Widespread metastases in the lungs and liver
3 (21)	Female	73	Recurrent bilateral hemothoraces accompanied by dyspnea	ERG (++), CD31 (++), D2-40 (podoplanin, co-expressed), Pleural epithelioid angiosarcoma	/	Palliative care	Multifocal brain metastases
4 (22)	Male	68	Sustained hemoptysis along with weight loss	CD31&ERG strongly positive, MNF116(-), Ki67 20% positive	/	Gemcitabine and Docetaxel	Partial remission of the lung tumor mass
5 (1)	Male	78	Cough accompanied by persistent hemoptysis	CD31 (+-), ERG & FLI strongly positive	/	Doxorubicin	Marked remission
6 (23)	Female	45		Epithelioid angiosarcoma	Untargeted mutation, including *RB1*, *BRAF*, *D594* and *TP53*	Paclitaxel	Marked remission
7 (24)	Female	79	Lung mass in the left upper lobe	CD31, factor VIII-related antigen, and vimentin strong positivity; Fli-1 and cytokeratin AE1/AE weak positivity.	/	/	Brain metastases, demise
8 (25)	Male	64	Shoulder and arm pain accompanied by chronic cough	CD31(+), CD34(+), Fli-1(+), AE1/AE3(+), CK7(+)	/	Palliative care	Died of brain and kidney metastasis
9 (26)	Male	62	Progressive dyspnea and bilateral massive hemothorax	Vimentin(++), WT1(++), CD31(+), Factor VIII-related antigen(+), CD34(+)	/	Surgery	Progression to death
10 (27)	Male	62	Constrictive pericarditis and recurrent pleural effusion	Fli-1(+) CD-31(+)	/	/	Died of heart attack

In the absence of clinical guidelines for epithelioid angiosarcoma, treatment decisions were grounded in extrapolated evidence from closely related neoplasms, including published data and clinical experience in soft tissue sarcoma (6) and non-small cell lung cancer. Comprehensive informed consent, detailing the investigational nature, potential risks, and anticipated benefits of each intervention, was obtained from both the patient and their legally authorized family representatives. These carefully considered therapeutic strategies are intended not only to address immediate clinical needs but also to generate actionable insights that may inform future prospective studies and treatment algorithms for this disease.

The rationale for combining drug-eluting bead bronchial arterial chemoembolization (DEB-BACE) (7) with systemic anlotinib therapy was supported by two convergent lines of evidence: first, transarterial chemoembolization (TACE) has demonstrated objective tumor responses and prolonged time-to-progression in case reports and small retrospective series involving hepatic epithelioid angiosarcoma (8); second, anlotinib—a multi-target tyrosine kinase inhibitor is approved in China for advanced soft tissue sarcoma (9). Following multidisciplinary tumor board review and shared decision-making—including detailed discussion of alternatives, uncertainties, and patient preferences—the combined modality was implemented for this case.

Regrettably, disease progression was observed on subsequent imaging. Given documented radiosensitivity in subsets of angiosarcomas (10), palliative radiotherapy remains a clinically appropriate option for symptom control in well-defined, accessible lesions. Accordingly, radioactive particle implantation was performed for selected subpleural lesions to reduce tumor burden and alleviate symptoms (11, 12). While this intervention achieved transient local control and symptomatic relief, it did not alter the trajectory of systemic disease progression on follow-up staging (13). This underscores the limitation of localized ablative approaches in the setting of widespread, biologically aggressive disease.

In accordance with the guidelines for soft tissue sarcoma, systemic chemotherapy has been employed in some cases documented in the literature (14). More specifically, doxorubicin, paclitaxel, docetaxel, and gemcitabine have all been utilized in the treatment of epithelioid angiosarcoma. Prior studies have reported that tumors in certain patients demonstrated regression following paclitaxel-based chemotherapy regimens. However, in the present case, tumor progression persisted despite the administration of paclitaxel, indicating an absence of chemotherapeutic sensitivity in this patient. Recently, immunotherapy has been increasingly investigated as a promising treatment option for rare tumors (15). However, in this specific case, likely due to the TMB-L/MSS status, the administration of the PD-1 inhibitor Tislelizumab was ineffective.

Previous studies have demonstrated that angiosarcoma exhibits molecular heterogeneity. It has been found that *MYC* gene amplification or *MYC* protein overexpression has been detected in a subset of angiosarcoma cases, which is helpful for diagnosis (14). Furthermore. In a molecular study of 34 angiosarcoma cases (16) investigated using targeted NGS, more than half of the cases harbored genetic alterations affecting the *MAPK* pathway, including mutations in *KRAS*, *HRAS*, *NRAS*, *BRAF*, *MAPK1*, and *NF1*, as well as amplifications in *MAPK1/CRKL*, *CRAF*, or *BRAF*. In prior studies, mutations associated with Pulmonary EA have been reported to involve *TP53*, *NRAS*, *MYC*, and *BRAF* (17)(as detailed in **Table 2**). Thus, targeted therapy has been recommended in some cases. However, this case represents the first documented instance of the *EML4-ALK* fusion in pulmonary EA.

The *ALK* gene was initially identified in anaplastic large cell lymphoma in 1994. Since then, the translocation and activation of *ALK* have been confirmed in various tumors. With advancements in sequencing technologies, *ALK* positivity has been detected in certain rare tumors. Notably, a broad spectrum of mesenchymal tumors may exhibit *ALK* gene rearrangements.

Among the four types of *EML4-ALK* fusions, the *EML4-ALK* Variant 1 (EX13:EX20) identified in this case represents the most prevalent fusion subtype. Furthermore, the *ALK* gene is capable of forming fusion genes with fusion partners such as *EML4*, *KIF5B*, *KLC1*, and *TFG (*18).

Malignant tumors harboring *ALK* rearrangements are relatively rare; however, *ALK*-targeted therapies for these tumors exhibit high efficacy. Therefore, the development of *ALK*-targeted therapies has progressed rapidly. Based on the previous researches (19), multiple generations of *ALK* inhibitors are clinically available: 1st-gen crizotinib (limited CNS penetration); 2nd-gen alectinib, ceritinib, brigatinib, and ensartinib (improved potency/CNS access); 3rd-gen lorlatinib (broad activity against resistance mutations, strong CNS penetration); and emerging 4th-gen agents (e.g., NVL-655) targeting compound mutations like G1202R/L1196M. Sequencing strategies increasingly rely on NGS—tissue-based or liquid biopsy (ctDNA)—to detect fusions, point mutations (e.g., F1174L, R1275Q), or amplifications across tumor types. *ALK* inhibitors function by binding to the ATP-binding site of *ALK*, thereby inhibiting tyrosine kinase activation and suppressing tumor cell proliferation and survival.

Recent literature reports a case where an *EML4-ALK* fusion NSCLC patient experienced significant tumor regression following neoadjuvant therapy with ensartinib (20). In a meta-analysis, the efficacy of eight *ALK* inhibitors was compared based on overall survival, progression-free survival, and objective response rate. Ensartinib demonstrated promising potential as a first-line systemic treatment option relative to other *ALK* inhibitors. Hence, in this clinical case, upon confirming the presence of the *EML4-ALK* fusion and determining the ineffectiveness of prior multiple lines of treatment, we selected ensartinib-based targeted therapy. The outcome was encouraging, providing a novel precedent for the application of *ALK*-targeted therapy in EA.

Like other TKIs, however, *ALK* inhibitors are constrained by acquired resistance. The underlying mechanisms include: (1) *ALK* kinase domain mutations (e.g., G1202R), (2) bypass track activation independent of ALK (e.g., MET/EGFR amplification, SHP2/MAPK signaling), (3) histological plasticity (e.g., SCLC transformation), and (4) microenvironmental shielding (e.g., CCL19/CCR7–PI3Kγ axis). Addressing these challenges will necessitate combinatorial multi-target inhibition, immunotherapy integration, or immune microenvironment reprogramming to restore treatment sensitivity and prolong therapeutic efficacy.

By presenting this case of pulmonary EA and reviewing related cases (**Table 2**), we propose that in rare tumor scenarios where standardized guidelines are lacking, the application of NGS for identifying therapeutic targets and developing personalized treatment plans can maximize patient benefits while avoiding ineffective interventions. Additionally, as more rare tumor cases undergo NGS, the resulting data will serve as valuable references for future treatment strategies.

## Data Availability

The raw data supporting the conclusions of this article will be made available by the authors, without undue reservation.
